# Nomogram for pre-procedural prediction of non-curative endoscopic resection in patients with early gastric cancer

**DOI:** 10.1007/s00464-023-09949-0

**Published:** 2023-02-28

**Authors:** So Young Han, Hong Jin Yoon, Jie-Hyun Kim, Hye Sun Lee, Jaeyoung Chun, Young Hoon Youn, Hyojin Park

**Affiliations:** 1 Department of Internal Medicine, Gangnam Severance Hospital, Yonsei University College of Medicine, 211 Eonjuro, Gangnam-Gu, Seoul, 135-720 Republic of Korea; 2 Department of Internal Medicine, Soonchunhyang University College of Medicine, 31 Sunchenonhyang 6-gil, Dongnam-gu, Cheonan, Republic of Korea; 3 Biostatistics Collaboration Unit, Yonsei University College of Medicine, Seoul, Republic of Korea

**Keywords:** Early gastric cancer, Submucosal endoscopic resection, Curative resection

## Abstract

**Background:**

Non-curative resection (non-CR) after endoscopic submucosal dissection (ESD) requires additional surgery due to the possibility of lymph node metastasis (LNM). Therefore, it is important to accurately predict the risk of non-CR to avoid unnecessary preoperative procedures. Thus, we aimed to develop and verify a nomogram to predict the risk of non-CR prior to ESD.

**Methods:**

Patients who underwent ESD for early gastric cancer (EGC) were divided into CR and non-CR groups based on the present ESD criteria. The pre-procedural factors, such as endoscopic features, radiologic findings, and pathology of the lesion, were compared between the groups to identify the risk factors associated with non-CR. A nomogram was developed using multivariate analysis, and its predictive value was assessed using an external validation group.

**Results:**

Among 824 patients, 682 were curative (82.7%) and 142 were non-curative (17.3%). By comparing two groups, endoscopic features including redness, whitish mucosal change, fold convergence, and large lesion size; histologic features such as moderately or poorly differentiated or signet ring cell carcinoma; and abnormal CT findings including non-specific lymph node enlargement and fold thickening were identified as significant predictors of non-CR. The nomogram was developed based on these predictors and showed good predictive performance in the external validation, with an area under the curve of 0.87.

**Conclusions:**

We developed a nomogram to predict the risk of non-CR prior to ESD. These predictive factors in addition to the existing ESD criteria can help provide the best treatment option for patients with EGC.

**Supplementary Information:**

The online version contains supplementary material available at 10.1007/s00464-023-09949-0.

For patients with early gastric cancer (EGC), endoscopic submucosal dissection (ESD) is an attractive treatment option to curatively remove tumors in a minimally invasive manner. There are well-established absolute and expanded indications for ESD set by the Japanese gastric cancer treatment guidelines to effectively treat cancer with minimal risk of lymph node metastasis (LNM) [1].

Physicians undertake various pre-procedural evaluations, such as endoscopy, abdominal computed tomography (CT), and endoscopic ultrasonography, to select only suitable cases for successful ESD. Despite these efforts, it is reported that approximately 16.1–21.4% of patients who receive ESD do not meet the indication of curative resection (CR) later and are considered non-curatively resected [2–4]. As up to 7.5–9.3% of non-curative resection (non-CR) tumors are known to have LNM [5, 6], patients with non-CR eventually undergo additive treatments, including repeated ESD or gastrectomy. Additive surgery after ESD poses a marked burden for patients both physically and financially. Thus, it is important for physicians to accurately predict the possibility of non-CR before ESD to avoid unnecessary procedures before surgery. Nonetheless, it is difficult to predict non-CR beforehand because it can only be confirmed by careful pathologic examination of the resected specimen.

Several previous studies have evaluated the predictive factors associated with non-CR and increased risk of LNM based on post-procedural histologic and surgical data [4, 7]. Kim et al., in their study, reported that age, tumor size, lymphatic invasion, depth of invasion, and histological differentiation were significant predictive factors for LNM [7]. However, there are very few studies on the prediction of non-CR solely based on pre-procedural clinicopathologic features, especially studies that incorporate endoscopic, pathologic, and radiologic data together. In reality, it is extremely important to predict the chance of non-CR based on the pre-procedural information because these are the only data that clinicians can obtain before deciding the treatment option for patients.

Thus, we aimed to investigate the pre-procedural features for predicting the risk of non-CR before ESD. We focused on endoscopic, radiologic, and pathologic features of the tumor, so as to depict the real-world clinical situation, by including the most frequently used pre-procedural diagnostic modalities.

## Patients and methods

### Patients

For our study, we retrospectively reviewed patients with EGC from two different hospitals: a group of patients for the development of the nomogram (“development set)” and another group for the external validation of our nomogram (“validation set”).

To develop the nomogram, we reviewed the data of 824 patients from Gangnam Severance Hospital who underwent ESD for EGC between 2010 and 2019. Patients aged > 19 years who underwent ESD for primary gastric cancer were included in the study. The patients were subsequently categorized into a CR group of 682 patients and a non-CR group of 142 patients based on their post-procedural histologic report. The criteria for the CR group were decided based on the Japanese gastric cancer treatment guidelines [8]. Unlike the Japanese guidelines’ definition of non-CR, where it considered non-CR for all lesions that do not meet the absolute or expanded criteria of CR, we only included selected definitions that we considered to be the inevitable sign of additive gastrectomy. The selected criteria for non-CR in our study included positive vertical margin, lympho-vascular invasion (LVI), submucosal invasion in undifferentiated-type EGC, and submucosal invasion > 500 μm in differentiated-type EGC. Moreover, cases of piecemeal resection or en bloc resection with horizontal margin involvement were not included in the non-CR group because these lesions can possibly be treated with secondary ESD.

The patients’ demographic information, including age and sex, and clinicopathologic information about tumor size, tumor location, gross appearance, depth of invasion, histology of the lesion, existence of LNM after additive surgery, endoscopic features of the lesions, and abnormal CT findings was collected.

For the validation set, we collected data from 129 patients at Soonchunhyang Hospital who met our study’s inclusion criteria. When classified according to our definition of CR and non-CR, 48 and 81 patients were categorized into the non-CR and CR groups, respectively. An identical set of data regarding patients’ demographic and clinicopathologic information was collected for this study.

Our study was approved by the Institutional Review Board of Yonsei University (Institutional Review Board no. 3-2022-0127), and all patient information was anonymized and de-identified before analysis.

### Data collection

For the analysis of pre-procedural features, we mainly focused on endoscopic, radiologic, and pathologic data.

**Fig. 1:**
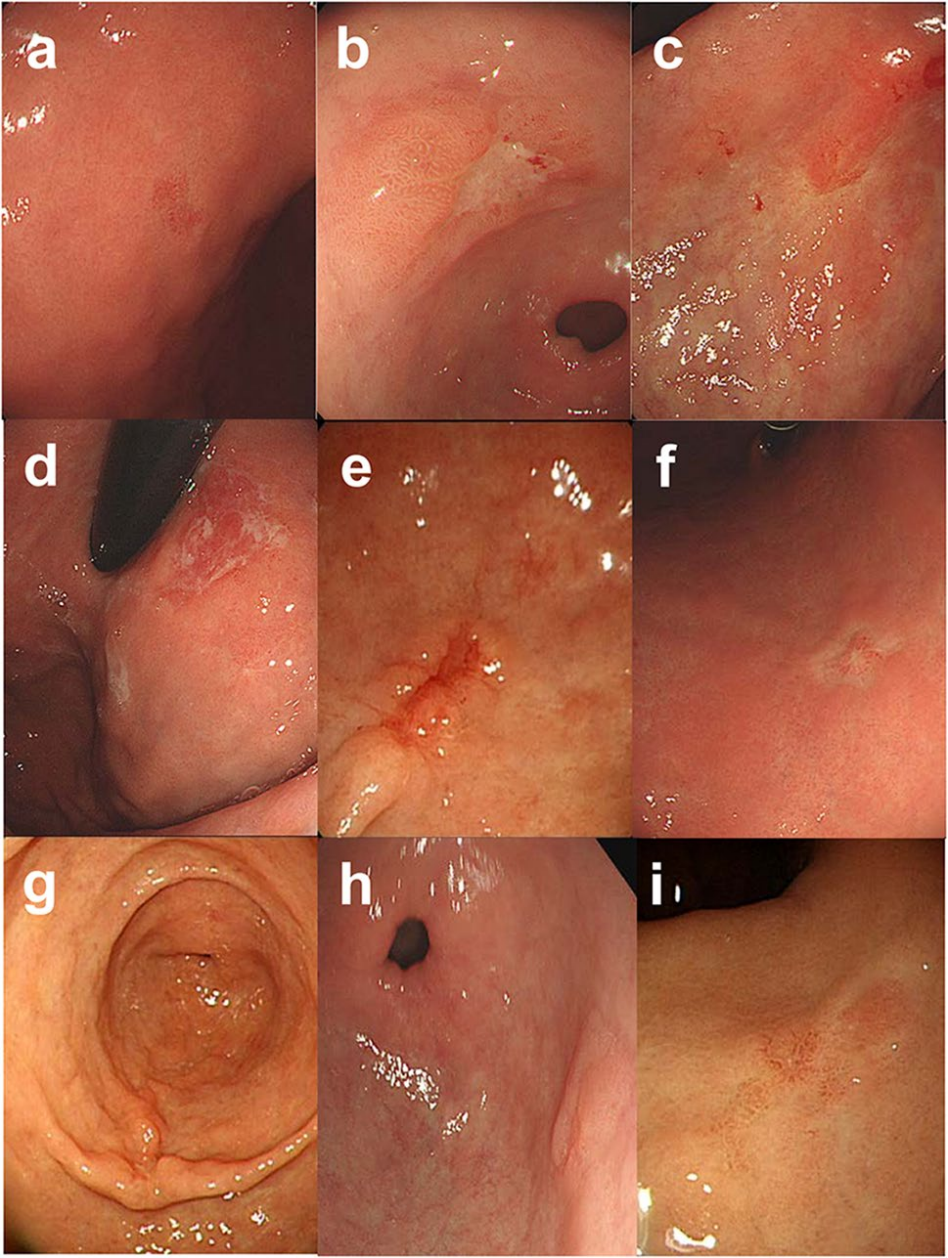
Endoscopic features of the early gastric cancer: **a** Redness, **b** Ulceration, **c** Scar, **d** Exudate, **e** Bloody discharge, **f** Whitish mucosal change, **g** Fold convergence, **h** Marginal nodularity, **i** Surface nodularity

For endoscopic analysis, all the patients’ pre-procedural endoscopic images were reviewed by two expert endoscopists from the Gangnam Severance Hospital (Fig. 1). Images were classified into the following nine categories: redness, ulceration, scar, exudate, bloody discharge, whitish mucosal change, fold convergence, marginal nodularity, and surface nodularity. These categories were determined based on the endoscopic features known to be related to mucosal and submucosal gastric cancers in previous studies [2, 9, 10]. Redness was defined as a reddish mucosal color change, which is similar to the regenerative epithelium, and whitish mucosal change was noted when the lesion was pale compared with the surrounding normal mucosa. Ulceration was diagnosed when the tumor lesion had a depressed and ulcerative surface. A scar was defined as a reddish or whitish scarring lesion. An exudate was noted when there was superficial whitish discharge surrounding the mucosa. Bloody discharge was defined when the lesion was friable and bled easily. Fold convergence was defined as thickened and merged gastric folds surrounding a lesion. Marginal nodularity and surface nodularity were noted when there was an uneven protruding lesion on the margin or surface of the tumor lesion.

For radiologic analysis, patients’ abdominopelvic CT images taken as part of the pre-procedural evaluation were used. They were carefully reviewed by an experienced radiologist from Gangnam Severance Hospital. We focused on abnormal CT findings of non-specific lymph node (LN) enlargement, mucosal thickening, or enhancement (Supplementary Fig. 1). Since LNs larger than 1 cm are generally considered markedly enlarged LNs with a high potential for malignancies, we only included cases with benign features, like LNs that were < 1 cm in size and had an oval or elongated shape [11, 12].

For pathological analysis, we classified the histology of the gastric tumors according to the World Health Organization classification system [13]. For the subgroup analysis in our study, we classified the lesions according to the Japanese classification system of gastric adenocarcinomas: differentiated-type (well-differentiated, moderately differentiated, and papillary adenocarcinoma) and undifferentiated-type (poorly differentiated adenocarcinoma and signet ring cell carcinoma) histology [14, 15].

### Statistical analysis

The continuous variables are described as mean values with standard deviations, and categorical variables are presented as numbers with percentages. We identified pre-procedural factors associated with non-CR primarily using univariate analysis and analyzed them using the chi-square test or Fisher’s exact test for categorical variables and Student’s t test for continuous variables. Variables that were significant in the univariate analysis were subsequently included in the multivariate analysis. Multivariate analysis was performed using multiple logistic regression, and nomograms were constructed based on the prediction model built using multivariate analysis. Later, the predictive abilities of our models were evaluated using the receiver operating characteristic (ROC) curve and calibration plot. For all our studies, a *p* value < 0.05 was considered statistically significant. SPSS (IBM Corp., Armonk, New York, USA) and R package version 4.0.5 were used for analysis.

## Results

### Baseline characteristics of the patients

The comprehensive baseline characteristics of the development and validation sets are presented in Table 1. The development set predominantly consisted of males with a mean age of 65.1 ± 11.0 years. The mean tumor size was 19.3 mm. The gross appearance of the lesions was classified according to the Japanese classification of gastric carcinoma, and the majority of the lesions (802 of 824 lesions) were flat (97.3%). A total of 663 patients had mucosal tumor invasion (80.4%); 705 patients had differentiated-type histology of EGC (85.6%), and 119 patients had undifferentiated-type histology (14.4%).

**Table 1:** Baseline demographic and clinicopathologic characteristics of the training and validation sets

Variables	Training set (*n* = 824)	Validation set (*n* = 129)
	*n* (%)	*n* (%)
Sex		
Male	604 (73.3)	87 (67.4)
Female	220 (26.7)	42 (32.6)
Age (year)		
Mean ± SD	65.1 ± 11.0	68.2 ± 9.06
Size (mm)		
Mean ± SD	19.3 ± 12.9	18 ± 10.9
Tumor location		
Upper 1/3	28 (3.4)	1 (0.8)
Mid 1/3	376 (45.6)	48 (37.2)
Lower 1/3	420 (51.0)	80 (62.0)
Gross appearance^b^		
Elevated	17 (2.1)	39 (30.2)
Flat	802 (97.3)	37 (28.7)
Depressed	5 (0.6)	53 (41.1)
Depth of invasion		
Mucosa	663 (80.4)	81 (62.8)
Submucosa	161 (19.6)	48 (37.2)
Histology^a^		
Differentiated	705 (85.6)	109 (84.5)
Undifferentiated	119 (14.4)	20 (15.5)
Non-CR group	142 (17.2)	48 (37.2)
Positive vertical margin	35 (4.2)	24 (18.6)
LVI	53 (6.4)	24 (18.6)
SM in UD-EGC^c^	40 (4.4)	16 (12.4)
≥ SM1 in D-EGC^d^	84 (10.1)	32 (24.8)
LNM after additive surgery	11 (1.3)	3 (2.3)
Endoscopic features		
Ulceration	117 (14.2)	17 (13.2)
Fold convergence	121 (14.7)	10 (7.8)
Redness	527 (64.0)	47 (36.4)
Exudate	366 (44.4)	36 (27.9)
Surface nodularity	322 (39.1)	87 (67.4)
Marginal nodularity	507 (61.5)	25 (19.4)
Scar	53 (6.4)	6 (4.7)
Blood discharge	58 (7.0)	17 (13.2)
Whitish mucosal change	87 (10.6)	11 (8.5)
Abnormal CT finding	193 (23.4)	31 (24.0)
Mucosal thickening	79 (9.6)	27 (20.9)
LN enlargement	99 (12.0)	3 (2.3)
Both	15 (0.2)	1 (0.1)

*EGC* early gastric cancer, *SD* standard deviation, *LN* lymph node, *LVI* lympho-vascular invasion, *CR* curative resection, *LNM* lymph node metastasis, *CT* computed tomography

^a^According to the Japanese classification

^b^According to the Paris classification

^c^Submucosal invasion in undifferentiated EGC

^d^Submucosal invasion > 500 μm in differentiated-type EGC

Of the 824 patients who underwent ESD, 142 were later classified into the non-CR group (17.2%). The reasons for non-CR were sub-analyzed: 35 (4.2%), 53 (6.4%), 40 (4.4%), and 84 (10.1%) patients had vertical margin involvement, LVI, submucosal invasion in undifferentiated-type EGC, and submucosal invasion > 500 μm, respectively. Among patients with non-CR who received additive surgery later, 11 (1.3%) were confirmed to have LNM by surgical pathology.

The endoscopic and radiologic features of these patients were reviewed. Abnormal CT findings were noted in 193 (23.4%) patients. Mucosal thickening was found in 79 patients (9.6%); LN enlargement, in 99 patients (12.0%); and both mucosal thickening and LN enlargement, in 15 patients (0.2%).

The clinicopathological features of the validation set were analyzed and are listed in Table 1. Validation set was also consisted of predominantly male patients with a mean age of 68.2 ± 9.06. The majority of the lesions (80 of 120 lesions) were located in lower third of the stomach. Of the 129 patients, 48 were later classified into the non-CR group (37.2%), showing higher rate of non-CR than the development set.

### Factors associated with non-CR

Univariate and multivariate analyses of pre-procedural features were performed on data from the development set to identify the variables associated with non-CR (Table 2). In univariate analysis, endoscopic features including redness, whitish mucosal change, fold convergence, bloody discharge, ulcer size > 20 mm, and gross appearance of the lesion; pathology of the lesion; and abnormal CT findings were identified as significant variables. Through our multivariate analysis, endoscopic features including redness (odds ratio [OR] 2.52; 95% confidence interval [CI] 1.54–4.12), whitish mucosal change (OR 2.17; 95% CI 1.17–4.03), fold convergence (OR 5.13; 95% CI 3.11–8.47), lesion size over 20 mm (OR 3.04; 95% CI 1.98–4.69), and elevated lesion (OR 1.85; 95% CI 1.10–3.14); pathology of moderately differentiated adenocarcinoma (OR 229; 95% CI 1.42–3.71), poorly differentiated adenocarcinoma (OR 11.61; 95% CI 5.70–8.32), or signet ring cell carcinoma (OR 3.60; 95% CI 1.55–8.32); and abnormal CT findings, including LN enlargement (OR 2.18; 95% CI 1.21–3.96), or the combination of fold thickening and LN enlargement (OR 4.62; 95% CI 1.33–16.1) were identified as predictive factors associated with non-CR (Table 2).

**Table 2:** Univariate and multivariate analyses of factors associated with non-curative resection

Variables	CR^c^ (*n* = 682)	Non-CR^d^ (*n* = 142)	Univariate	Multivariate	
	*n* (%)	*n* (%)	*p* value	Odds ratio (95% CI)	*p* value
Endoscopic feature					
Redness	419 (61.4)	108 (76.1)	0.001	2.52 (1.54–4.12)	< 0.001
Whitish mucosal change	63 (9.2)	24 (16.9)	0.012	2.17 (1.17–4.03)	0.014
Fold convergence	70 (10.3)	51 (35.9)	< 0.001	5.13 (3.11–8.47)	< 0.001
Blood discharge	41 (6.0)	17 (12.0)	0.012		
Ulcer	78 (11.4)	39 (27.5)	< 0.001		
Size ≥ 20 mm	194 (28.4)	77 (54.2)	< 0.001	3.04 (1.98–4.69)	< 0.001
Gross appearance^a^			< 0.001		
Flat	339 (49.7)	47 (33.1)		Reference	
Elevated	142 (20.8)	45 (31.7)		1.85 (1.10–3.14)	0.021
Depressed	201 (29.5)	50 (35.2)		1.38 (0.83–2.27)	0.212
Pathology^b^			< 0.001		
Well differentiated	380 (55.7)	39 (27.5)		Reference	
Moderately differentiated	226 (33.1)	60 (42.3)		2.29 (1.42–3.71)	0.001
Poorly differentiated	29 (4.3)	32 (22.5)		11.61 (5.70–23.6)	< 0.001
Signet ring cell	47 (6.9)	11 (7.7)		3.60 (1.55–8.32)	0.003
Abnormal CT finding			< 0.001		
Fold thickening	55 (8.1)	24 (16.9)		1.54 (0.80–2.97)	0.199
LN enlargement	76 (11.2)	23 (16.2)		2.18 (1.21–3.96)	0.010
Both	8 (1.2)	7 (4.9)		4.62 (1.33–16.1)	0.016

*CI* confidence interval, *LN* lymph node, *CR* curative resection, *CT* computed tomography

^a^According to the Paris classification

^**b**^World Health Organization classification

^c^CR curative resection

^d^Non-CR non-curative resection

Subsequently, we sub-analyzed the data based on lesion histology. Patients were grouped based on differentiation of gastric tumors as differentiated and undifferentiated Univariate and multivariate analyses were performed for both groups. The results of the comprehensive analyses for both groups are listed in Tables 3 and 4.

**Table 3:** Factors associated with non-curative resection in differentiated-type histology

Variables	CR^c^ (*n* = 606)	Non-CR^d^ (*n* = 99)	Univariate	Multivariate	
	*n* (%)	*n* (%)	*p* value	Odds ratio (95% CI)	*p* value
Endoscopic features					
Bloody discharge	32 (5.3)	11 (11.1)	0.025	2.37 (1.07–5.25)	0.033
Redness	379 (62.5)	72 (72.7)	0.05	1.95 (1.15–3.33)	0.014
Whitish mucosal change	42 (6.9)	15 (15.2)	0.005	2.69 (1.31–5.53)	0.007
Fold convergence	65 (2.8)	39 (16.2)	< 0.001	5.13 (3.05–8.62)	< 0.001
Size ≥ 20 mm	194 (32.0)	47 (47.5)	0.003		0.060
Gross appearance^a^			0.017		
Elevated	135 (22.3)	35 (35.4)			
Flat	301 (49.7)	35 (35.4)			
Depressed	170 (28.1)	29 (29.3)			
Pathology^b^			< 0.001		
Well differentiated	380 (62.7)	39 (39.4)		Reference	
Moderately differentiated	226 (37.3)	60 (60.6)		2.45 (1.52–3.96)	< 0.001
Abnormal CT finding	123 (20.4)	39 (39.4)	< 0.001	2.35 (1.44–3.83)	0.001
Fold thickening	46 (7.6)	15 (15.2)		2.10 (1.03–4.29)	0.042
LN enlargement	70 (11.6)	18 (18.2)		2.49 (1.32–4.69)	0.005
Both	7 (1.2)	6 (6.1)		7.83 (2.23–27.5)	0.001

*CI* confidence interval, *LN* lymph node, *CR* curative resection, *CT* computed tomography

^a^According to the Paris classification

^**b**^World Health Organization classification

^c^CR curative resection

^d^Non-CR non-curative resection

**Table 4:** Factors associated with non-curative resection in undifferentiated-type histology

Variables	CR^a^ (*n* = 76)	Non-CR^b^ (*n* = 43)	Univariate	Multivariate	
	*n* (%)	*n* (%)	*p* value	Odds ratio (95% CI)	*p* value
Endoscopic features					
Redness	40 (52.6)	36 (83.7)	0.001	6.04 (2.02–18.0)	0.001
Fold convergence	5 (6.7)	12 (27.9)	0.002	6.54 (1.72–24.9)	0.006
Pathology^c^					
Poorly differentiated	29 (38.2)	32 (74.4)	< 0.001	Reference	
Signet ring cell	47 (61.8)	11 (25.6)	< 0.001	0.27 (0.11–0.67)	0.005
Gross appearance					
Elevated	7 (9.2)	10 (23.3)	0.025		
Flat	38 (50.0)	12 (27.9)			
Depressed	31 (40.8)	21 (48.8)			
Abnormal CT finding			0.425		
Fold thickening	9 (11.8)	9 (20.9)			
LN enlargement	6 (7.9)	5 (11.6)			
Both	1 (1.3)	1 (2.3)			

*CI* confidence interval, *LN* lymph node, *CR* curative resection, *CT* computed tomography

^a^CR curative resection

^b^Non-CR non-curative resection

^c^World Health Organization classification

^d^According to the Paris classification

In multivariate analysis of the patients with differentiated-type histology (Table 3), endoscopic features including bloody discharge (OR 2.37; 95% CI 1.07–5.25), redness (OR 1.15; 95% CI 1.15–3.33), whitish mucosal change (OR 2.69; 95% CI 1.31–5.53), and fold convergence (OR 5.13; 95% CI 3.05–8.62); pathology of moderately differentiated adenocarcinoma (OR 2.45; 95% CI 1.52–3.96); and abnormal CT findings, including fold thickening (OR 2.10; 95% CI 1.03–4.29), LN enlargement (OR 2.49; 95% CI 1.32–4.69), or a combination of both (OR 7.83; 95% CI 2.23–27.5), were identified as significant variables to predict non-CR.

There were fewer variables associated with non-CR in the multivariate analysis of undifferentiated-type histology (Table 4). Only endoscopic features including redness (OR 6.04; 95% CI 2.02–18.0) and fold convergence (OR 6.54; 95% CI 1.72–24.9) were identified as significant predictive factors associated with non-CR. Signet ring cell carcinoma was more common in the CR group and showed a negative correlation with non-CR (OR 0.27; 95% CI 0.11–0.67).

### Construction of nomograms

**Fig. 2:**
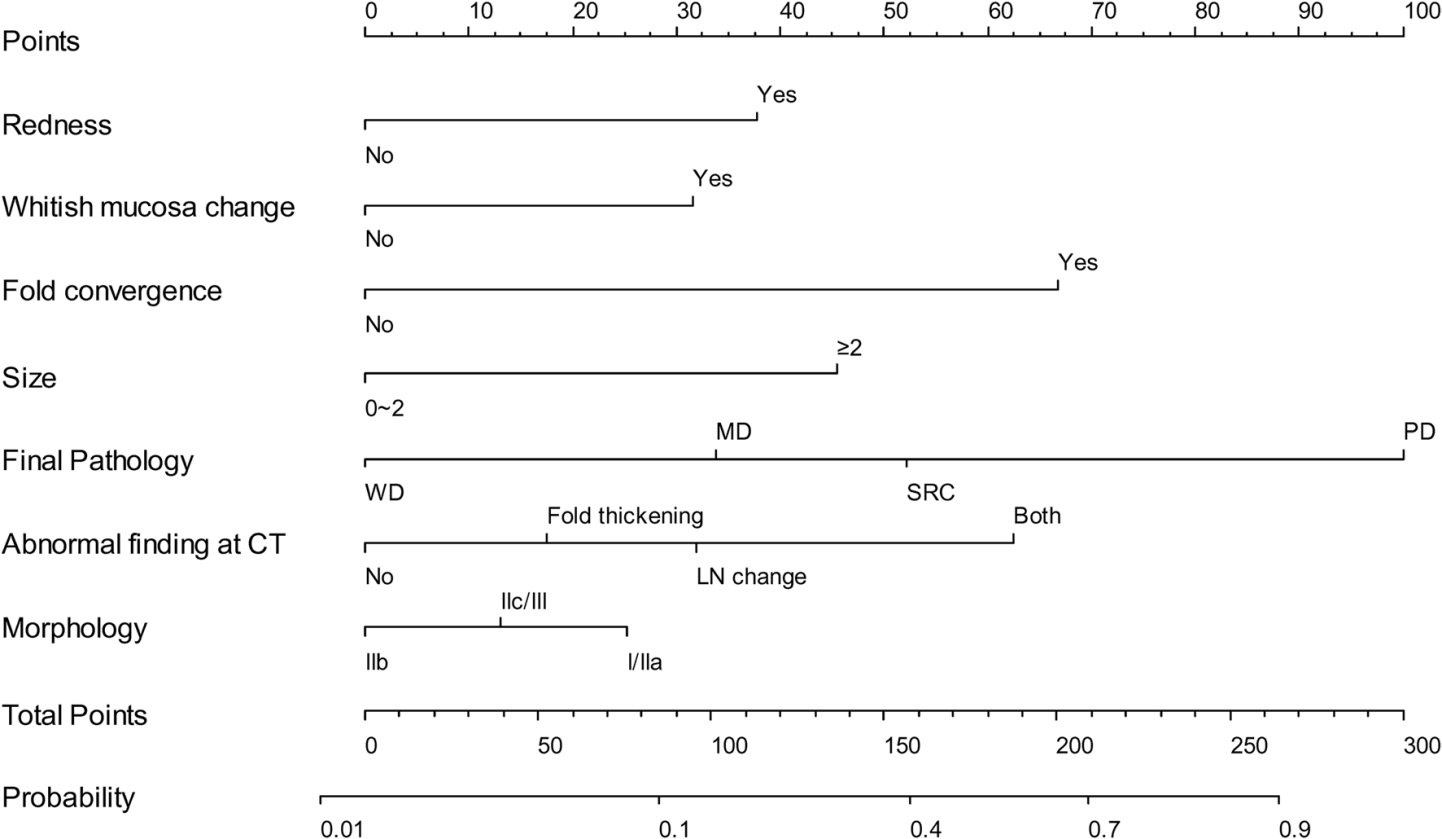
Nomogram predicting the risk of non-curative resection prior to endoscopic resection. *WD* well-differentiated, *MD* moderately differentiated, *SRC* signet ring cell carcinoma, *PD* poorly differentiated, *CT* computed tomography, *LN* lymph node

Based on the predictive variables obtained from the multivariate analysis, we developed a nomogram to predict the risk of non-CR using a logistic regression model (Fig. 2). Points were provided for each predictive variable. They were weighed differently in accordance with the calculated odds ratio, and the sum of all points was located on a total point scale. It was then vertically correlated with the probability scale to obtain the probability of non-CR. Pathology of the lesion and fold convergence contributed the most to the model. A real-world example of the nomogram is presented in Supplementary Fig. 2, where the patient’s predicted probability of non-CR calculated by our model was 0.52. The patient’s post-ESD pathologic report revealed non-CR due to vertical margin involvement and LVI. The patient was later reported to have metastasis in 1 of 58 regional LNs in a post-surgical pathologic report.

**Fig. 3:**
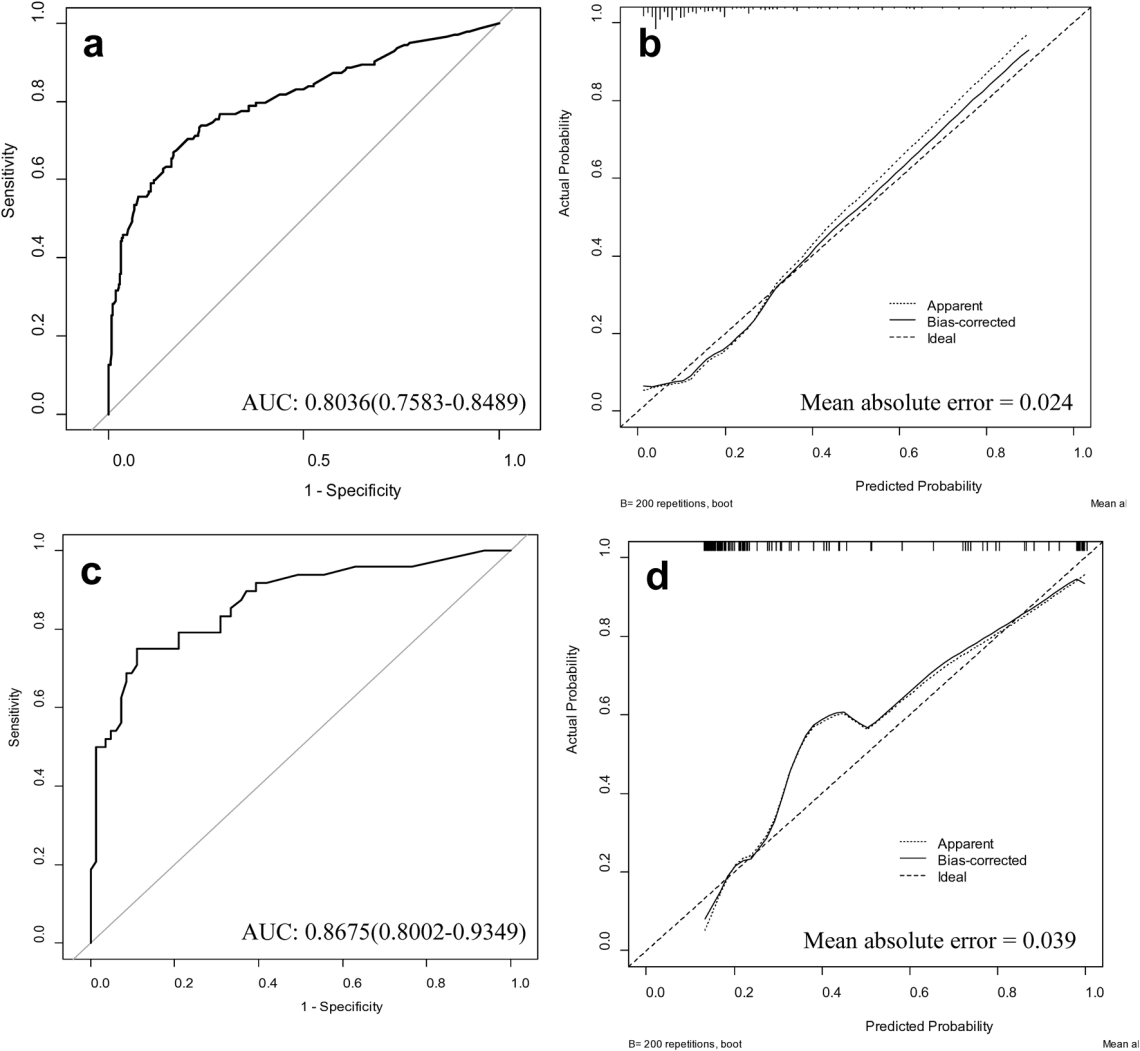
Receiver operating characteristics (ROC) curve and calibration plot of the development set and external validation set: **a** Receiver operating characteristics (ROC) curve of the development set, **b** Calibration plot of the development set, **c** Receiver operating characteristics (ROC) curve of the validation set, **d** Calibration plot of the validation set. *AUC* area under the curve

An ROC curve was constructed to assess the performance of our nomogram. The area under the curve (AUC) was 0.8036 (95% CI 0.7583–0.8489), establishing the good predictive ability of our model (Fig. 3). Figure 3 also shows the calibration plot of the nomogram, where the x-axis shows the predicted probability of non-CR by our nomogram, and the y-axis shows the actual probability. The bias-corrected calibration curve (solid line) was close to the ideal reference line (dashed line). This demonstrated a good fit between the predicted and actual results with a mean absolute error of 0.024. We also developed additional nomograms based on our sub-analysis. The nomograms constructed based on tumors with differentiated-type histology (Supplementary Fig. 3) and undifferentiated-type histology (Supplementary Fig. 4), as well as their ROC curves and calibration plots, are presented in our Supplementary Fig. 5. The AUC of the ROC curve was 0.7664 (95% CI 0.7098–0.8231) for differentiated-type histology and 0.8012 (95% CI 0.7235–0.879) for undifferentiated-type histology. Both substantiated the good predictive ability of our model.

### Validation of the nomogram

External validation of our nomogram was performed using a set of 129 patients from Soonchunhyang Hospital who underwent ESD for EGC. The ROC curve was plotted to assess the performance of our nomogram, which showed a high AUC of 0.8675 (95% CI 0.8002–0.9349). This indicated the good predictive ability of our nomogram for non-CR (Fig. 3). The calibration plot of the nomogram also demonstrated a good fit with a mean absolute error of 0.039. For the evaluation of nomograms of the sub-analysis groups, the nomogram for differentiated-type histology showed an acceptable AUC for the ROC curve of 0.8631 (95% CI 0.7753–0.9509) (Supplementary Fig. 6). However, we were unable to obtain satisfactory results for the AUC of the ROC curve for undifferentiated-type histology. This was mainly because our external validation set consisted of a very small number of tumors with undifferentiated-type histology.

## Discussion

Gastric cancer is one of the most frequently diagnosed cancers globally, with over one million new cases in 2020 and an estimated 769,000 deaths [16]. Endoscopic resection is known to have several advantages over gastrectomy, such as shorter hospital stay, reduced overall postoperative morbidity, and lower medical costs [17, 18].

Even with extensive pre-procedural evaluation, some patients are required to receive additive treatment. Additive surgery after endoscopic resection is a great burden to patients, especially considering that a large number of patients with gastric cancer are older adults. As Akagi et al. reported in their study that gastrectomy after ESD can lead to a higher risk of surgical complications, partly due to its increased probability of intra-abdominal adhesions [19]. To overcome these limitations, we developed a nomogram to predict non-CR using pre-procedural clinicopathological information obtained from patients with EGC.

Several recent studies have attempted to evaluate risk factors for non-CR. Nam et al. mainly focused on endoscopic features and concluded that female sex, large lesion size, longer procedural time, location of cancer, and endoscopic findings, such as ulceration, nodularity, and depression, were predictive factors [2]. However, most of these studies were limited to the analysis of endoscopic features of the lesion, and several studies lacked external validation. Another study by Ma et al. developed a nomogram to predict non-CR by analyzing various variables, including endoscopic features, resection method, and postoperative histology findings [20]. However, this study has limitations in its practical application because some of the predictive variables in the nomogram are only available post-procedurally. To the best of our knowledge, our research is the only study to develop a predictive nomogram by analyzing pre-procedural risk factors obtained from endoscopic, pathologic, and radiologic data. The current National Comprehensive Cancer Network (NCCN) guidelines for gastric cancer recommend various diagnostic modalities, such as CT scans of the chest, abdomen, and pelvis, to decide on pretreatment staging [21]. Therefore, the biggest strength of our study is that we developed a predictive model using data from various diagnostic modalities that are strictly available pre-procedurally. In addition, we have externally validated the performance of our model.

In our study, we identified endoscopic features including redness, whitish mucosal change, fold convergence, and lesion size > 20 mm; pathology of moderately differentiated adenocarcinoma and poorly differentiated adenocarcinoma or signet ring cell carcinoma; and abnormal CT findings, including LN enlargement or the combination of fold thickening and LN enlargement, as predictive factors associated with non-CR. To date, CT scans have mainly been used to evaluate the nodal involvement of cancer; however, our study results show that even non-specific CT findings, such as LN enlargement or fold thickening, may play an important role in predicting non-CR. We also constructed nomograms based on these predictive variables and evaluated their predictive ability using ROC curves and calibration plots. The AUC of the ROC curve for both the development and external validation sets showed good predictive abilities of our models.

Our study has several limitations. First, since the number of cases with undifferentiated-type histology was relatively small in the external validation group (20 of 129 patients), we were not able to achieve satisfactory AUC results for our external validation for the undifferentiated-type histology group. Further evaluations using a larger study size will provide more consistent results in future. Second, because not all of the patients’ primary diagnoses were confirmed in a single center, we analyzed the histology of the final resected specimens instead of the initial forceps biopsy data. Since the histologic discrepancy between forceps biopsy and endoscopic resection specimen is not reported to be high, (approximately 4.4–10.7%) [22–24], we believe our result can be applied to data with forceps biopsy as well. Lastly, endoscopic features characterized in our study may seem vulnerable to interobserver variation. However, in reality, endoscopic diagnosis and treatment plans are determined largely based on the opinion of the endoscopist who evaluates the lesion. Our study has helped to establish concordant indices among endoscopists for prediction of non-CR by identifying characteristic features. We believe application of artificial intelligence can resolve current limitations, and our team is actively pursuing improvements through multicenter studies.

In conclusion, we developed a nomogram and externally validated it to predict non-CR in patients with EGC using various pre-procedural data. We believe that our model can help physicians and patients to choose the best treatment options and avoid unnecessary procedures before surgery.

## Supplementary Information

Below is the link to the electronic supplementary material.

Supplementary file1 (PDF 220 KB)
