# Supplementary material for: Nomogram for pre-procedural prediction of non-curative endoscopic resection in patients with early gastric cancer
Source: Surg Endosc. 2023 Feb 28;37(6):4594–603. doi: 10.1007/s00464-023-09949-0 (PMC10234883; doi:10.1007/s00464-023-09949-0)
Supplement: Supplementary file 1 — Supplementary file1 (PDF 220 KB) [file 464_2023_9949_MOESM1_ESM.pdf]

Original Article

Nomogram for pre-procedural prediction of non-curative endoscopic resection in patients with early gastric cancer

So Young Han[1*], Hong Jin Yoon[2], Jie-Hyun Kim[1], Hye Sun Lee[3], Jaeyoung Chun[1], Young Hoon Youn[1], HyoJin Park[1]

[1]Department of Internal Medicine, Gangnam Severance Hospital, Yonsei University College of Medicine, Seoul, Korea

[2]Department of Internal Medicine, Soonchunhyang University College of Medicine, 31 Sunchenonhyang 6-gil, Dongnam-gu, Cheonan, Republic of Korea

[3]Biostatistics Collaboration Unit, Yonsei University College of Medicine, Seoul, Korea

Short running head: Nomogram for prediction of non-CR

Correspondence to: Jie-Hyun Kim, MD, PhD

Address: Department of Internal Medicine, Gangnam Severance Hospital, Yonsei University College of Medicine, 211 Eonjuro, Gangnam-gu, Seoul, Korea, 135-720

Phone: 82-2-2019-3505

Fax: 82-2-3463-3882

E-mail: otilia94@yuhs.ac

Supplementary Fig 1. Abnormal CT findings of the early gastric cancer: **a** Fold thickening; **b** Non-specific lymph node enlargement

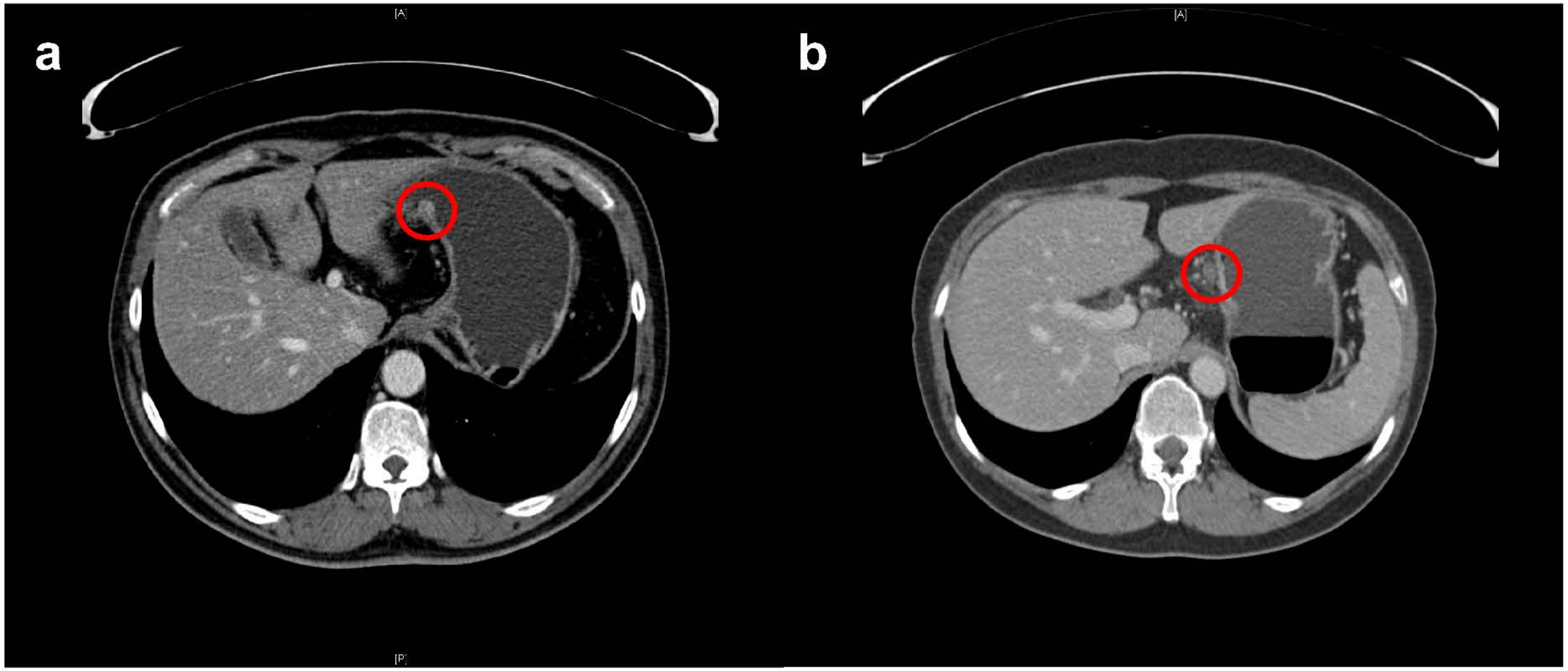

Supplementary Figure 2. Real-world example of nomogram use : A real-world application of the nomogram is depicted herein. Each variable was assessed, and the predicted probability of non-CR was calculated using our model. The patient's post-ESD pathologic report revealed non-CR due to vertical margin involvement and lymphovascular invasion.

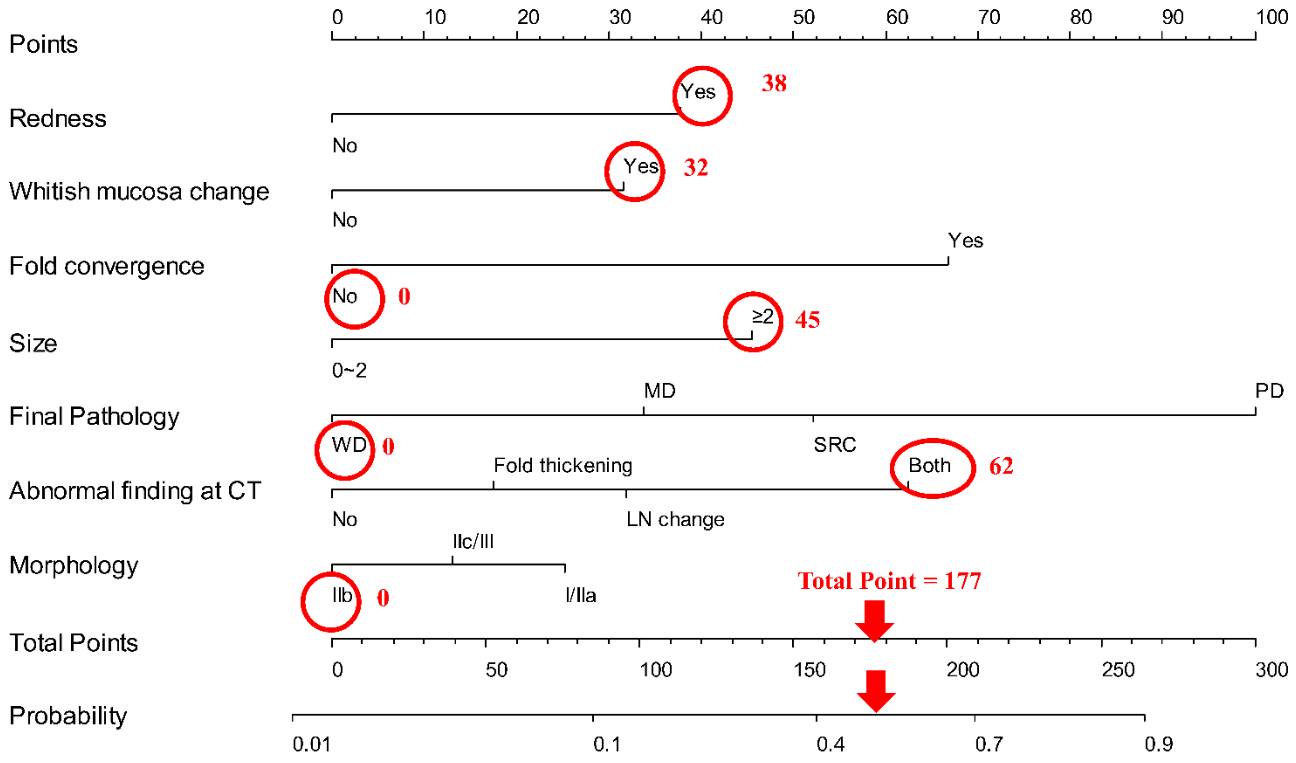

Abbreviations: *WD* well-differentiated; *MD* moderately differentiated; *SRC* signet ring cell carcinoma; *PD* poorly differentiated; *CT* computed tomography; *LN* lymph node

Supplementary Figure 3. Nomogram predicting the risk of non-curative resection in differentiated-type histology

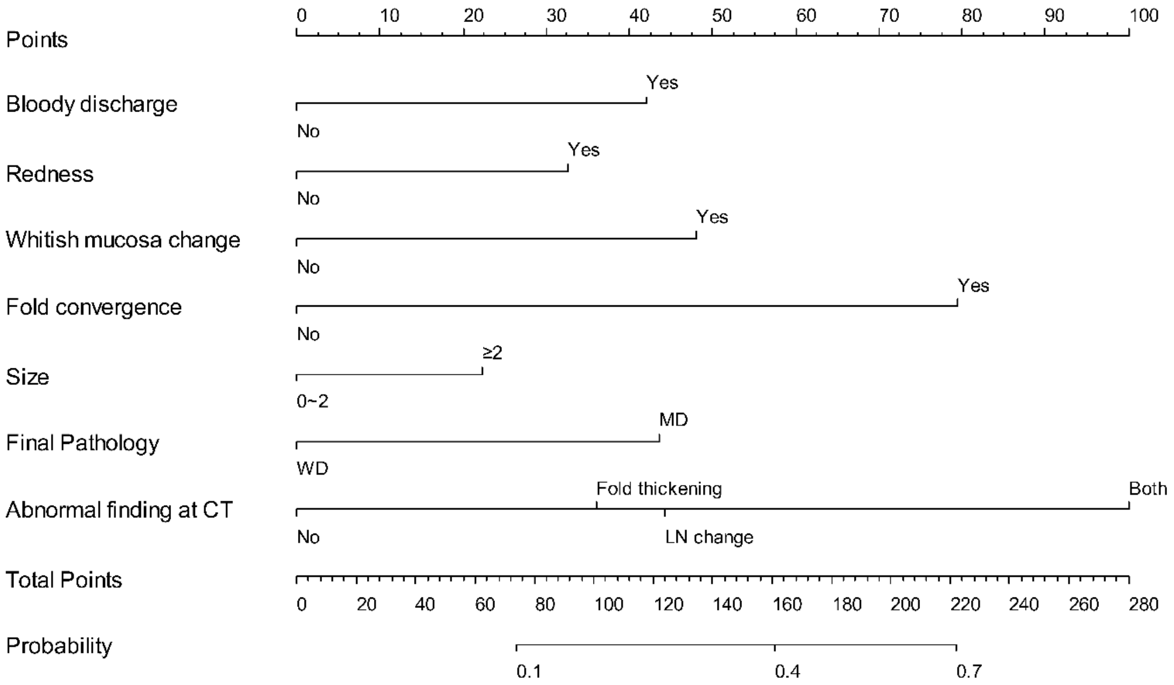

Abbreviations: *WD*, well-differentiated; *MD*, moderately differentiated; *CT* computed tomography; *LN* lymph node

Supplementary Figure 4. Nomogram predicting the risk of non-curative resection in undifferentiated-type histology

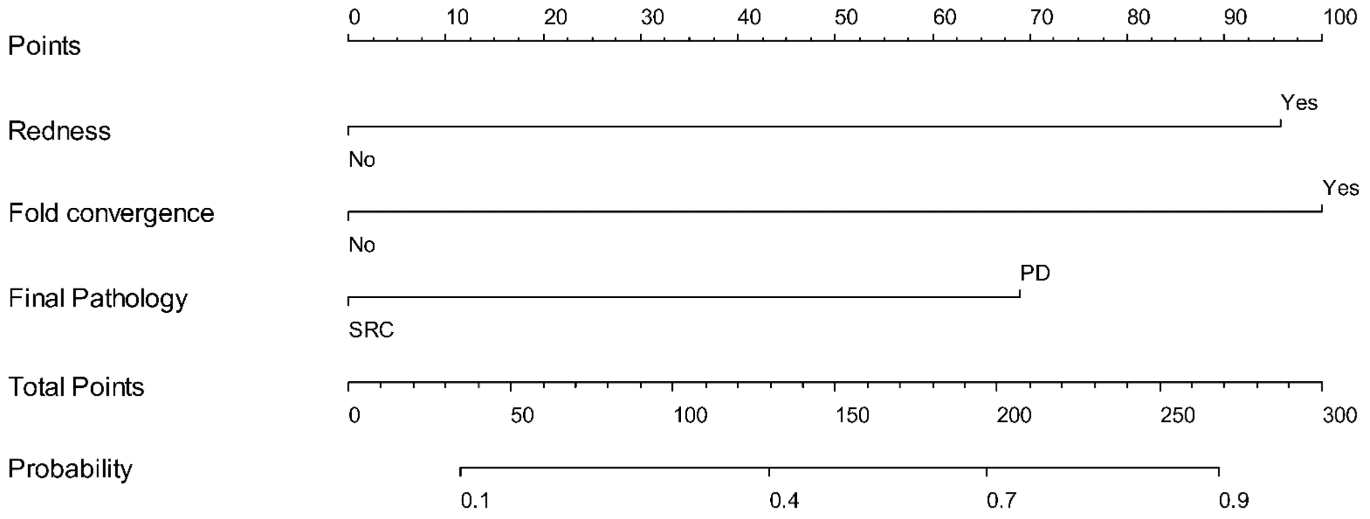

Abbreviations: *SRC* signet ring cell carcinoma; *PD* poorly differentiated

Supplementary Figure 5. Receiver operating characteristic (ROC) curves of differentiated- and undifferentiated-type histology in the development set: **a** Receiver operating characteristics (ROC) curve of the differentiated-type histology; **b** Receiver operating characteristics (ROC) curve of the undifferentiated-type histology

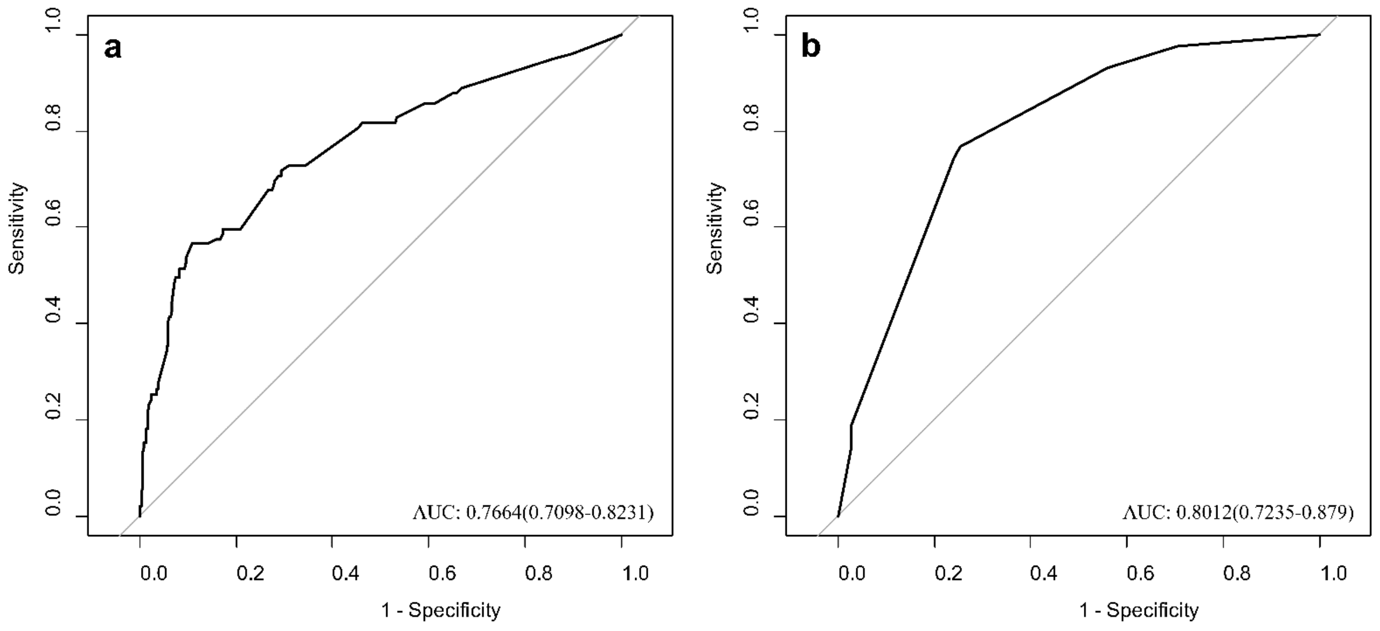

Abbreviation: *AUC* area under the curve

Supplementary Figure 6. Receiver operating characteristic (ROC) curves of differentiated- and undifferentiated-type histology in the external validation set: **a** Receiver operating characteristics (ROC) curve of the differentiated-type histology; **b** Receiver operating characteristics (ROC) curve of the undifferentiated-type histology

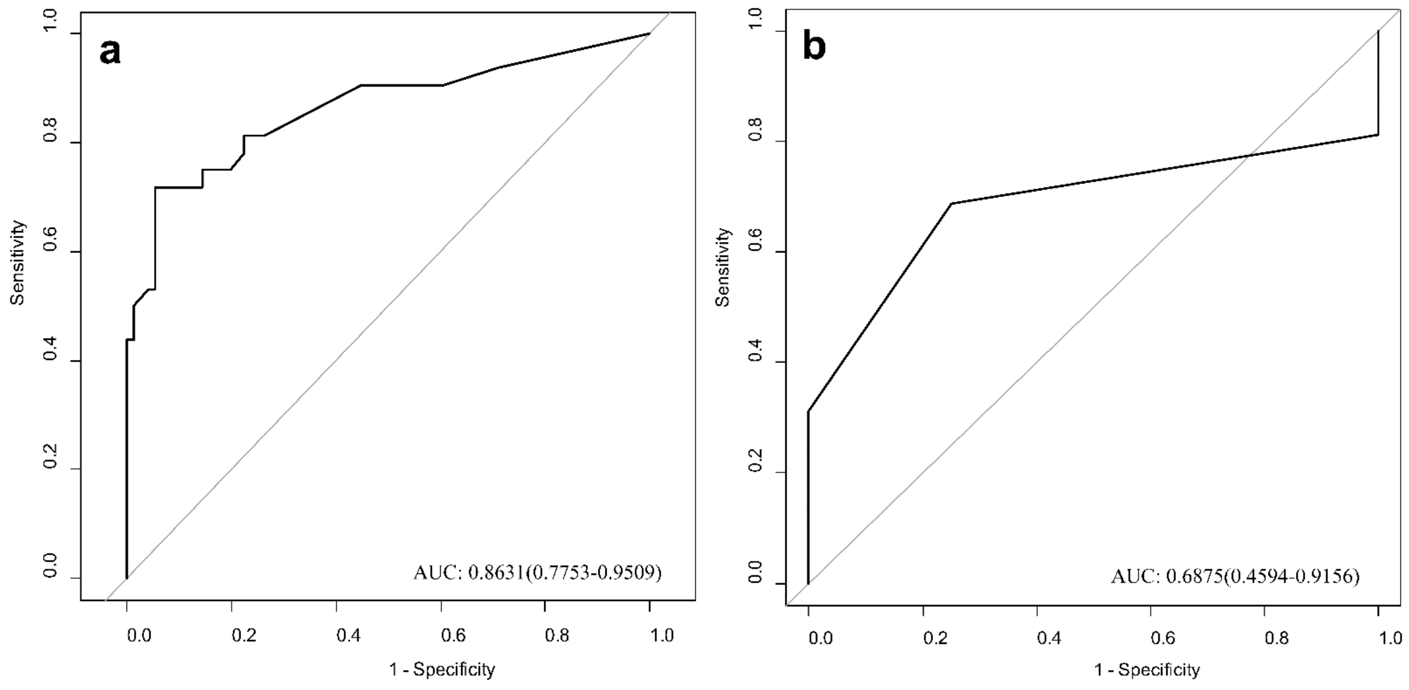

Abbreviation: *AUC* area under the curve
